# Role of mitochondria-associated endoplasmic reticulum membranes in insulin sensitivity, energy metabolism, and contraction of skeletal muscle

**DOI:** 10.3389/fmolb.2022.959844

**Published:** 2022-10-07

**Authors:** Bianca Nieblas, Perla Pérez-Treviño, Noemí García

**Affiliations:** ^1^ Escuela de Medicina y Ciencias de la Salud, Tecnologico de Monterrey, Monterrey, Nuevo León, México; ^2^ Experimental Medicine and Advanced Therapies, The Institute for Obesity Research, Tecnologico de Monterrey, Monterrey, Nuevo León, México

**Keywords:** mitochondria-associated ER membranes (MAMs), insulin resistance, mitochondrial skeletal muscle, mitochondrial dysfunction, obesity, mitochondrial subpopulations

## Abstract

Skeletal muscle has a critical role in the regulation of the energy balance of the organism, particularly as the principal tissue responsible for insulin-stimulated glucose disposal and as the major site of peripheral insulin resistance (IR), which has been related to accumulation of lipid intermediates, reduced oxidative capacity of mitochondria and endoplasmic reticulum (ER) stress. These organelles form contact sites, known as mitochondria-associated ER membranes (MAMs). This interconnection seems to be involved in various cellular processes, including Ca^2+^ transport and energy metabolism; therefore, MAMs could play an important role in maintaining cellular homeostasis. Evidence suggests that alterations in MAMs may contribute to IR. However, the evidence does not refer to a specific subcellular location, which is of interest due to the fact that skeletal muscle is constituted by oxidative and glycolytic fibers as well as different mitochondrial populations that appear to respond differently to stimuli and pathological conditions. In this review, we show the available evidence of possible differential responses in the formation of MAMs in skeletal muscle as well as its role in insulin signaling and the beneficial effect it could have in the regulation of energetic metabolism and muscular contraction.

## Introduction

Mitochondrial dysfunction and endoplasmic reticulum (ER) stress have been described as key mechanisms in the development of insulin resistance (IR) in the skeletal muscle (SM) system (Zamora and Villena, 2014). Both organelles independently regulate fundamental cellular functions; mitochondria as the main energy-producing organelles and the ER as the site of synthesis of proteins and lipids, as well as with regard to its role in calcium homeostasis (Namba, 2019; Zhang et al., 2021). Besides their biological roles, it has been described that mitochondria and ER are structurally and functionally connected through contact sites named mitochondria-associated ER membranes (MAMs), also known as mitochondria–ER contact sites (MERCs) (Tubbs et al., 2018; Gordaliza-Alaguero et al., 2019; Yang et al., 2020; Zhang et al., 2021). MAMs are constituted by resident proteins with a specific function, as well as cytosolic proteins, forming multiprotein complexes such as IP3R1-GRP75-VDAC1, Mfn1-Mfn2, and Fis-Bap31 complexes, among others (Table 1 describes some proteins identified at MAMs in different cell types and Figure 2A illustrate the main proteins at MAMs identified in the SM) (Giacomello and Pellegrini, 2016; Gordaliza-Alaguero et al., 2019; Townsend et al., 2020; Barazzuol et al., 2021).

**TABLE 1:** Main proteins and protein complexes involved in MAMs and IR in skeletal muscle and other tissues.

**Protein**	**Cellular Localization**	**Intrinsic Function**	**Complex Tether**	**Complex Function**	**Tissue**	**Changes associated to insulin resistance**	**References**
**DRP1** (Dynamin-related protein 1)	Cytosol/recruited to mitochondria during fission	Mitochondrial fission	DRP1-CTRP1	Recruitment of DRP1 at mitochondria and interaction with ER through CTRP1	MEFs cells (Mouse embryonic fibroblasts)	Not studied	Sonn et al. (2021)
						DRP1-Mfn1	Colocalization at tethering sites and coordinate the fusion and fission events	U-2 OS (epithelial osteosarcome cells) and HeLa cells	Not studied	Abrisch et al. (2020)
**Fis1** (Mitochondrial fission 1 protein)	Outer mitochondrial membrane	Mitochondrial fission	FIS1-BAP31	Tether mitochondria to ER/SR for transfer of apoptotic signals	HeLa and HEK293T cells (immortaliz ed human embryonic kidney cells)	Not studied	Iwasawa et al. (2011)
**Grp75** (Glucose- regulated protein)	Cytosol	Chaperone	IP3R1-Grp75- VDAC1	Mediated conformational coupling between the IP3R1 and VDAC1	Liver tissue	Reduced IP3R1-Grp75 (decrease of tethering)	Tubbs et al. (2014)
					Skeletal muscle	Experimental overexpression restored the tethering and the alterations of insulin signaling	Tubbs et al. (2018)
					Skeletal muscle	Overexpression and increase of tethering. Silencing restored insulin-stimulated AKT signaling	Thoudam et al. (2019)
**IP3R1** (Inositol 1,4,5- trisphosphate receptor)	ER/SR	SR Ca^2+^ release channel 1	IP3R1-Grp75- VDAC1	Tether mitochondria to ER/SR for mitochondrial Ca^2+^ uptake	Liver tissue and skeletal muscle	Reduced IP3R1- VDAC1 and IP3R1-Grp75 (decrease of tethering)	Tubbs et al. (2014)
							Tubbs et al. (2018)
					Liver tissue and skeletal muscle	Overexpression related to increase of tethering. Silencing restored insulin-stimulated AKT signaling	Arruda et al. (2014) Thoudam et al. (2019)

						IP3R1-CypD	Tether mitochondria to ER/SR	Liver tissue	KO of CypD reduced tethering and promotes alterations of insulin signaling	Tubbs et al. (2014)
			**MCU** (Mitochondrial Ca^2+^ uniporter)	Inner mitochondrial membrane	Ca^2+^ transfer into the mitochondrial matrix	Coordinates its function with that of the IP3R1 Grp75- VDAC1	Ca^2+^ transfer into the mitochondria	Liver tissue	Overexpression related to increase of tethering and elevated Mitochondrial Ca^2+^	Arruda et al. (2014)
**Mfn1** (Mitofusin 1)	Outer mitochondrial membrane	Mitochondrial Fusion	Mfn1	Mfn1 location at MAMs sites indicate the mitochondrial fusion site	U-2 OS and HeLa cells	Not studied	Abrisch et al. (2020)
**Mfn2** (Mitofusin 2)	Outer mitochondrial membrane	Mitochondrial fusion	Mfn2-Mfn2 Mfn2-Mfn1	Regulation of tethering mitochondria to ER/SR	Embryonic fibroblasts, HeLa cells, SH-SY5Y cells (neuroblastoma cell line), MEFs and skeletal muscle	Not studied	de Brito and Scorrano, (2008)
							Han et al. (2021)
							Filadi et al. (2015)
							Castro Sepulveda et al. (2021)
							Marini et al. (2020)
					Skeletal muscle	Mfn2 downregulation. Mfn2 overexpression related to improve of insulin sensitivity	Kong et al. (2013).
										Skeletal muscle	Experimental overexpression restored IP3R1- VDAC1, the tethering and the alterations of insulin signaling	Tubbs et al. (2018)
					**Opa1** (Optic atrophy protein 1)	Inner mitochondrial membrane	Mitochondrial fusion	Not reported	Regulation of tether mitochondria to ER/SR	Fibroblast	Not studied	Cartes- Saavedra et al. (2022)
**PACS-2** (Phosphofurin acidic cluster sorting protein 2)	ER and mitochondria	Membrane trafficking	PACS-2-Bid	Regulator of MAMs and apoptosis	MCF7, HeLa, and A7 melanoma	Not studied.	Simmen et al. (2005)
					Liver tissue	Overexpression related to increase of MAMs and mitochondrial Ca^2+^ overload	Arruda et al. (2014)
					**PDK4** (Pyruvate dehydrogenase kinase 4)	Mitochondrial matrix	Mitochondrial serine/threonine kinase	IP3R1-Grp75- VDAC1-PDK4	Regulation of tether mitochondria to ER/SR	Skeletal muscle	Overexpression and increase of tethering	Thoudam et al. (2019)
**VDAC1** (Voltage- dependent anion channel 1)	Outer Mitochondrial Membrane	Exchange of metabolites and Ca^2+^ between mitochondria, ER and cytosol	IP3R1-Grp75- VDAC1	Regulation of Ca^2+^ transfer from ER to mitochondrial intermembrane space	Liver tissue and skeletal muscle	Reduced VDAC1 expression and IP3R1-VDAC1 (decrease of tethering)	Tubbs et al. (2014)
							Tubbs et al. (2018)
					Skeletal muscle	Overexpression and increase of tethering	Thoudam et al. (2019)

Abbreviations CTRP1, C1q/TNF-related protein 1; CypD, Cyclophilin D; ER, endoplasmic reticulum; KO, knockout; SR, sarcoplasmic reticulum.

Mitochondria and ER contacts were first described in rat liver (Shore and Tata, 1977), by electron microscopy, which was later followed by biochemical evidence, such as phospholipid transfer between the two organelles (Jain and Zoncu, 2022). More recently, it was found in hepatocytes that at least one in four mitochondria is associated with the ER, covering ∼4%–11% of its surface (Giacomello and Pellegrini, 2016). In 1987, MAMs were identified in the *vastus lateralis* of the Japanese meadow frog (Ogata and Yamasaki, 1987), a muscle constituted by type I and type II fibers (Staron et al., 2000). Three fiber types have been identified in SM with distinct metabolic properties; type I fibers have an oxidative metabolism, type IIa fibers are identified as more glycolytic, and type IIx fibers present a combination of both oxidative and glycolytic metabolism (Zierath and Hawley, 2004) (Fisher et al., 2017). More recently, an exhaustive analysis carried out in striated muscle, which included glycolytic/oxidative SM and cardiac muscle, revealed that mitochondrial contact sites with ER are present in >97% of all mitochondria analyzed in striated muscles and are involved in Ca^2+^ flux regulation. Another interesting result was the identification of contact sites between lipid droplets and mitochondrial networks, these being more frequent in oxidative than glycolytic muscles; in addition, lipid droplet–connected mitochondria showed fewer contact sites with the sarcoplasmic reticulum (SR), suggesting a lower capacity for Ca^2+^ handling according to the muscle type (Bleck et al., 2018). These findings indicate that MAMs could be modified according to cell metabolic state (Giacomello and Pellegrini, 2016).

It has been stated that MAMs function as a platform of communication between ER and mitochondria predominantly for Ca^2+^-mediated signaling, lipid biosynthesis, and control of the energetic process. Bioenergetic capacity, in turn, is related to the quality of the mitochondrial population; consequently, it had been proposed that MAMs could regulate mitochondrial quality control, which includes mitochondrial biogenesis, mitochondrial dynamics, and mitophagy, as well as apoptosis (Tubbs and Rieusset, 2017; Tubbs et al., 2018; Yang et al., 2020). On the other hand, MAM-related processes have been reported to interact with insulin signaling and to be tightly linked with insulin sensitivity in insulin-dependent organs, that is, SM; therefore, disruptions of its integrity are closely associated with the loss of both insulin action and secretion, having an impact on muscle response to changes in nutrient availability and inducing IR (Cheng et al., 2020; Zhang et al., 2020). In addition, an important factor is the SM phenotype heterogeneity through the different subcellular locations in which mitochondria can be found and possibly interact with the ER. Therefore, in this review, we show evidence of MAM constitution in the SM and how those contact sites regulate key processes, such as muscle contraction, mitochondrial function, apoptosis, and insulin sensitivity.

### Type of fibers and mitochondrial population of skeletal muscle: Mitochondria-associated endoplasmic reticulum membrane density and insulin sensitivity

Mitochondria from SM are organized in a highly complex reticular network that allows energy distribution to supply the cell demand (Kuznetsov et al., 2006; Glancy et al., 2015; Vincent et al., 2019). Based on their localization, three mitochondrial populations have been identified in muscle cells: 1) subsarcolemmal mitochondria (SSM), 2) intermyofibrillar mitochondria (IMF), and 3) perinuclear mitochondria (PNM) (Kuznetsov et al., 2006). SSM reside in clusters beneath the sarcolemma; they have an irregular arrangement and have larger dimensions than IMF but represent a lower volume proportion (Kuznetsov et al., 2006; Ferreira et al., 2010; Glancy et al., 2015); they are generally globular with a few branches (Vincent et al., 2019). IMF are located between myofibrils, have a higher surface area–to-volume ratio, are smaller, and have a more homogenous size (Ferreira et al., 2010; Willingham et al., 2021). IMF are organized in pairs at both sides in red fibers or next to the Z-line of each sarcomere in white fibers (Ogata and Yamasaki, 1997) (Vendelin et al., 2005). On the other hand, biochemical differences between mitochondrial populations include the distinct protein composition, lipids, and oxidative capacity. Among them, proteins such as the mitochondrial Ca^2+^ uniporter complex (MCU) and translocase of the outer mitochondrial membrane (TOM20) are expressed in a homogenous way through the muscle cell (Díaz-Vegas et al., 2018) and proteins associated with oxidative phosphorylation (OXPHOS) (Ferreira et al., 2010), as well as MICU1 (protein regulator of MCU), seem to be only distributed in the IMF subpopulation (Díaz-Vegas et al., 2018). In addition, differences between SSM and IMF populations have been found in the production or use of proton-motive force for ATP production related to the differential expression of OXPHOS proteins. Proteins associated with mitochondrial proton-motive force production (complex IV) (Glancy et al., 2015) and cytochrome c (cyt-c) (Díaz-Vegas et al., 2018) have been found to be predominantly expressed in the cell periphery, in the SSM, while proteins that use the proton-motive force for ATP production (complex V) are more expressed in the cell interior near contractile machinery in the IMF (Glancy et al., 2015). Regarding lipid content, cardiolipin content is higher in SSM than in IMF (Cogswell et al., 1993); it is the signature phospholipid of mitochondrial membranes, primarily found in the IMM, and is important for ATP generation by stabilizing electron transport chain (ETC) complexes and regulates processes such as mitochondrial dynamics, protein import, and mitophagy (Keenan et al., 2020).

These morphological and biochemical characteristics have been useful in distinguishing how each subpopulation regulates the functional capacity of mitochondria. IMF have a higher respiratory chain complex activity and are proposed to be specialized in energy production for contractile function; besides, their higher surface area–to-volume ratio allows the diffusion of ATP from mitochondria to the myofibrillar ATPase (Ferreira et al., 2010; Willingham et al., 2021), while SSM has a higher oxidative state which correlates with significantly higher Ca^2+^ levels in this subpopulation (responsible for higher mitochondrial respiration *via* activation of certain mitochondrial dehydrogenases) (Kuznetsov et al., 2006). In addition, SSM presents higher connectivity in the soleus muscle than in the gastrocnemius, constituted by fiber types I and II, respectively (Kuznetsov et al., 2006; Willingham et al., 2021). Compared to the IMF, an abundant SSM population is observed in lean subjects (Ritov et al., 2005). In obesity and T2D, SSM is preferentially affected, observed as a reduced density and reduction in the electron transport chain activity and oxidative capacity (Ritov et al., 2005; Lionetti et al., 2007) Therefore, in the regulation of metabolism, SSM seems to be involved in fatty acid oxidation, glucose transport, propagation of insulin signaling, or other signaling cascades important to insulin action (Ritov et al., 2005).

In SM, mitochondrial population and mitochondrial density can be adapted according to the metabolic demands (Howald et al., 1985). This plasticity of SM is also accompanied by the change in the fiber type ratio under different physiological and pathological conditions (Howald et al., 1985; Oberbach et al., 2006; Fisher et al., 2017). Type I fibers are those that present a higher mitochondrial density (Howald et al., 1985) and higher glucose-handling capacity related to higher expression of proteins for glucose transport and oxidation such as insulin receptor (INSR), glucose transporter type 4 (GLUT4), and hexokinase II (Albers et al., 2015). Even though the behaviour of mitochondrial populations can be different according to the fiber type, both IMF and SSM showed a higher capacity of fatty acid oxidation in fiber type I (Koves et al., 2005). Under exercise conditions, which is related to major insulin sensitivity, mitochondrial density, the population of SSM, and type I–to–type II ratio are increased, indicating higher oxidative capacity (Howald et al., 1985). A similar, positive correlation between type I fiber and insulin sensitivity has been observed in premenopausal women (Fisher et al., 2017). On the contrary, a lower type I–to–type II ratio is related to decreased insulin sensitivity, observed in obesity, metabolic syndrome, and T2D (Oberbach et al., 2006; Stuart et al., 2013; Albers et al., 2015). In addition, differences in the distribution of MAMs can be observed between the different fiber types. In glycolytic muscles which present a lower type I–to–type II ratio, there is observed a higher disposition of MAMs than that in the oxidative muscles (Bleck et al., 2018). Therefore, under that evidence, we propose that muscle with a lower proportion of type I fibers could present higher or altered disposition of contact sites or MAMs as well as those whose alteration could be related to mitochondrial dysfunction, ER stress, and, consequently, IR on metabolic disruption conditions such as obesity, metabolic syndrome, and T2D.

### Impaired insulin signaling by metabolic disruptions on skeletal muscle

**FIGURE 1:**
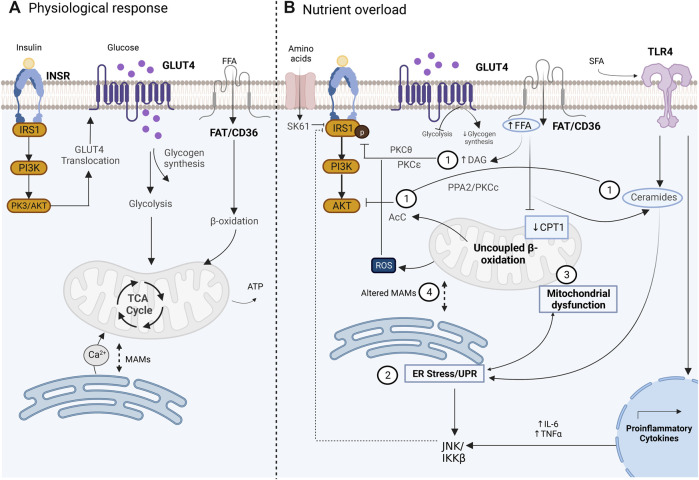
Glucose uptake in the skeletal muscle cell under physiological conditions mediated by the insulin pathway **(A)** Altered disrupted glucose uptake during insulin resistance pathology related to an overnutrition state and the main related contributors. **(B)** 1) lipid intermediates such as DAG, ceramides, and AcC which have been associated with altered activity of IRS1 and AKT. 2) UPR activation during ER stress with a not clear subjacent mechanism, but proposals include JNK activation which affects insulin signaling. 3) Mitochondrial dysfunction is related to altered fatty acid oxidation and increased ROS production. 4) Altered MAMs that affect the signal transduction between ER and mitochondria. Image created with BioRender.com.

In SM, when circulatory glucose is elevated, the insulin-regulated IRS1-PI3K-AKT pathway is activated to promote glucose uptake and maintain glucose homeostasis. The signaling begins with insulin binding to its receptor (INSR) which leads to the activation and recruitment of signaling proteins such as the insulin receptor substrate (IRS), the IRS-1 being the most relevant for SM, recruitment of phosphoinositide-3-kinase (PI3K), and protein kinase B (PKB/AKT). AKT activation mediates the translocation and fusion of GLUT4 to the plasma membrane, allowing glucose to enter the cell for its utilization and storage, through glycolysis and glycogen synthesis, respectively, both pathways being stimulated by insulin signaling (Figure 1A) (Boulinguiez et al., 2017; Petersen and Shulman, 2018; Barazzuol et al., 2021). IR is the tissue-reduced response to insulin, where normal circulating insulin levels are not enough to lower blood glucose levels, a condition that disrupts glucose and lipid homeostasis (Petersen and Shulman, 2018). Insulin signaling importantly regulates glucose homeostasis and lipid metabolism in SM, the liver, and white adipocytes; nevertheless, SM is the main tissue responsible for blood glucose uptake induced by insulin during the postprandial state (DeFronzo et al., 1981). Therefore, IR in SM importantly impacts blood glucose levels with consequences of altered insulin signaling for the whole body and is considered an important metabolic alteration related to obesity, which precedes T2D development (DeFronzo and Tripathy, 2009; Petersen and Shulman, 2018).

IR study dates back to 1960 (Yalow and Berson, 1960), and since that date, multiple studies had investigated the underlying mechanisms of IR. Currently, IR is considered a multifactorial condition, whose mechanisms continue to be studied and debated. The initial hypothesis in the 60s, the glucose fatty-acid cycle, postulated that IR resulted from an inhibition of glycolysis due to an increase in fatty acid oxidation in SM (Randle et al., 1963). In the 80s, human euglycemic-insulin clamp studies demonstrated that glycogen synthesis is the main non-oxidative pathway that follows the absorbed glucose in SM in normal subjects (DeFronzo et al., 1981), which was later confirmed and demonstrated to also occur in T2D (Shulman, et al., 1990). Nevertheless, in T2D, it was observed that glycogen synthesis is significantly reduced, which led to the postulation that defects in the glycogen synthesis pathway had an important role in the impairment of the insulin sensitivity observed in T2D (Shulman, et al., 1990). Subsequently, different *in vivo* studies in humans demonstrated that reduced insulin sensitivity and reduced glycogen synthesis are related to the impairment of glucose transport (Bonadonna et al., 1993; Cline et al., 1999; Shulman, 2000) due to altered translocation of GLUT4 to the plasma membrane (Garvey et al., 1998). Currently, it is well established that altered translocation of GLUT4 is a consequence of disruption of the insulin-regulated IRS1-PI3K-AKT pathway, where downregulation or defective activity of INRS, IRS1, PI3K, and AKT can lead to altered insulin sensitivity in SM with the consequence of reduced glucose uptake (Petersen and Shulman, 2018). Multiple factors have been implicated in the pathogenesis of IR in SM, such as lipotoxicity, inflammation, mitochondrial dysfunction, and ER stress (Figure 1B); all of them are intrinsically related, which complicates their individual study and understanding (Muoio and Newgard, 2008; Petersen and Shulman, 2018) and, thus, the MAMs’ role in insulin sensitivity regulation.

#### Lipid intermediates in skeletal muscle and insulin resistance

Accumulated evidence indicates that the overnutrition state, observed in human obese subjects and diverse animal models of high-fat diet (HFD), is a relevant factor for IR development (Petersen and Shulman, 2018). In this scenario, lipid over storage in adipose tissue leads to lipid accumulation in the liver and muscle which induces a toxic state in tissues, known as lipotoxicity, in muscle due to low expression of CPT1 (Koves et al., 2008). Impairment of insulin signaling by intramyocellular lipids (IMCLs) is related to the location, composition, and turnover of lipid intermediates such as diacylglycerols (DAGs), ceramides, and acylcarnitines (AcCs), whose role has been a subject of extensive study and debate over the last decades (Brøns and Grunnet, 2017). Some of the proposed mechanisms of IR development mediated by IMCL that have been described are 1*) the role of DAGs in the inhibition of IRS*. Activation of diverse isoforms of protein kinases C (PKC) such as PKC-θ and PKC-ε by DAGs are relevant events for insulin signaling disruption in SM and the liver, respectively. In SM, PKC-θ phosphorylate IRS proteins in serine residues, which in turn blocks the phosphorylation of tyrosine residue and therefore, impair PI3K activation (Gassaway et al., 2018; Petersen and Shulman, 2018; Kolczynska et al., 2020). In this context, it has been observed that IRS can also be inactivated by the kinases JNK and IKK-β, which are central transducers of the innate immunity activated in response to different stress signals such as infection, inflammatory cytokines, ROS, and ER stress and are therefore considered as the more relevant kinases for the IR understanding related to inflammation (Yuan et al., 2001; Solinas and Becattini, 2016). In addition, IRS can be inactivated by the kinase S6K1 under conditions of amino acid overload related to an overnutrition state (Tremblay et al., 2007). Therefore, the evidence suggests that the IRS protein is a central target for the regulation of insulin signaling in which different modulators converge, but the mechanisms by which it occurs have not been fully understood. 2) *The inhibition of AKT is mediated by accumulated ceramides.* The *novo* synthesis of ceramides can be stimulated under different stress conditions; under overnutrition, saturated fatty acids (SFAs) can induce inflammation through the activation of toll-like receptor 4 (TLR4), which is related to the induction of the *novo* synthesis of ceramides (Holland et al., 2011; Ritter et al., 2015), and the increase of the proinflammatory interleukin synthesis, such as TNF-alfa or IL-6 (Jani et al., 2021). Ceramides are related to IR through two proposed key mechanisms, the dephosphorylation of AKT mediated by the protein phosphatase 2A (PPA2) and by the activation of the protein kinase Cç (PKCç) which reduces the affinity of AKT for phosphoinositides, preventing its translocation to the plasma membrane (Figure 1B) (Xia et al., 2020). Despite the evidence of IR induced by ceramides, ceramide accumulation is not always observed in different models of IR caused by lipids; therefore, the role of ceramides in this context remains unclear (Petersen and Shulman, 2018). 3) *AcC accumulated by incomplete fatty acid oxidation*. In lipid oxidation, AcC is formed during fatty acid translocation to mitochondria for its β-oxidation; nevertheless, accumulated AcC are markers of an incomplete β-oxidation since they are overproduced by a mismatch between β-oxidation and TCA cycle flux related to an oversupply of mitochondrial lipid demand. The mechanisms of IR mediated by AcC have not been elucidated, but some hypotheses focus on the increase in ROS, the direct effect on AKT phosphorylation, and the activation of inflammation through NFκB (Ritter et al., 2015; Petersen and Shulman, 2018).

#### Skeletal muscle endoplasmic reticulum stress and insulin resistance

Increased demand for nutrient utilization which is observed in IR related to an overnutrition state has been associated with altered function of ER and mitochondria, particularly, the development of ER stress and mitochondrial dysfunction (Petersen and Shulman, 2018). ER or SR is a network of tubules and flattened sacs that extend throughout the cytosol, which is the place of the synthesis and folding of proteins, synthesis of steroids and lipids, and storage of Ca^2+^ in specialized domains (Schwarz and Blower, 2016). Under different stress inductors, such as overdemand of secretory proteins, heat shock, and mitochondrial calcium [mt (Ca^2+^)] overload, among others, the folding capacity of ER can be altered, leading to the accumulation of unfolded or misfolded proteins which disrupt ER homeostasis, considered ER stress (Walter and Ron, 2022). Consequently, the unfolded protein response (UPR) is activated to sustain the protein folding capacity of the ER. Three sensor proteins are the main mediators of the UPR: the protein kinase R (PKR)-like endoplasmic reticulum kinase (PERK), inositol-requiring protein 1a (IRE1a), and activating transcription factor 6 (ATF6), which represent three branches of the UPR activating transcriptional response through the transcriptional factors ATF6(N), XBP1, and ATF4, respectively (Walter and Ron, 2022). Sustained ER stress with consequent prolongated UPR activity indicates an exceeding of the capacity of the ER to sustain the protein folding demand, which induces an apoptotic response (Walter and Ron, 2022). The first finding of ER stress being linked to IR dates to a 2004 study in a murine HFD and a genetic obesity model, where increased expression of the chaperon GRP78 and increased phosphorylation of PERK, both indicators of ER stress, were found in liver and adipocyte tissues. Importantly, reduced insulin signaling through activation of JNK, which leads to inactivation of IRS1, was found. Nevertheless, all these changes were not observed in SM (Özcan et al., 2004). Currently, several studies have shown a link between UPR activation and IR in SM. In this context, induction of ER stress by lipid oversupply has been observed *in vivo* in HFD models in mice and *in vitro* treatment of free fatty acids (FFA) in C_2_C_12_ muscle cells. In these studies, increased expression of GRP78 and phosphorylation of PERK, IRE1a, ATF4, and CHOP have indicated ER stress, a condition alleviated by exercise or Omega-3 polyunsaturated fatty acid (n-3 PUFA) treatment (Deldicque et al., 2010; Tachtsis et al., 2020; Zhang et al., 2020). Nevertheless, in human SM biopsies of healthy subjects exposed to an HFD, the ER stress markers were unchanged despite a 50% increase in IMCL observed in SM, which suggests that ER stress does not play an important role in the onset of glucose intolerance, but it is not ruled out that in later stages, ER stress can be implicated (Deldicque et al., 2011).

#### Is mitochondrial dysfunction the cause or consequence of insulin resistance in skeletal muscle?

Mitochondria is the core organelle for ATP production and is involved in the regulation of important cellular processes such as metabolism, mitochondrial ROS generation, cellular death by apoptosis, cellular calcium homeostasis, and inflammation. Mitochondria modulate their content, location, activity, and shape to supply an appropriate response according to the cellular demand; loss of these capabilities is considered mitochondrial dysfunction (Diaz-Vegas et al., 2020). Mitochondrial dysfunction has been determined as an important component of IR according to the evidence obtained in multiple studies. In this regard, SM of subjects with obesity and T2D showed smaller mitochondria than that of lean subjects, reduced mitochondrial content, and reduced mitochondrial activity (indicated by the measure of the NADH oxidoreductase, succinate oxidase activity, or mitochondrial respiration) (Kelley et al., 2002; Ritov et al., 2005; Mogensen et al., 2007). In addition, lean subjects with IR and offspring of T2D patients showed reduced mitochondrial oxidative phosphorylation in muscle (analyzed *in vivo* by magnetic resonance spectroscopy), reduced AKT activation related to IRS1 phosphorylation, and increased IMCL not related to an increase in lipolysis in adipose tissue, suggesting that dysregulated intramuscular fatty acid metabolism is an important component of IR in a normal nutrition state related to an inherited mitochondrial dysfunction (Petersen et al., 2004; Morino et al., 2005; Befroy et al., 2007.) In a contradictory way, no significant differences were observed in the mitochondrial activity of T2D subjects when O_2_ flux capacity analyzed by respirometry was normalized with the mitochondrial content (Boushel et al., 2007). Similarly, in HFD rat models, an increase in mitochondrial content and lipid oxidation were observed, suggesting that decreased mitochondrial activity is not a direct link to IR (Holloszy, 2013). Therefore, the consensus suggests that multiple mitochondrial defects (changes in activity, content, or morphology) can be observed in IR, but the mechanisms that underlie them and whether this is a direct cause of IR or a consequence are not known with certainty. Currently, mitochondrial ROS has been postulated as an important link between mitochondrial dysfunction and IR. The SM of HFD mice and obese subjects showed an increase in mitochondrial H_2_O_2_ production and the scavenging of H_2_O_2_ confers protection against IR (Petersen and Shulman, 2018). In liver and adipose tissue, mitochondrial ROS have been found to be related to IRS1 inactivation by serine residue phosphorylation, leading to disrupted GLUT4 translocation (Potashnik et al., 2003; Al-Lahham et al., 2015; Sangwung et al., 2020).

### Role of mitochondria-associated endoplasmic reticulum membranes in the Ca^2+^ homeostasis of skeletal muscle

**FIGURE 2:**
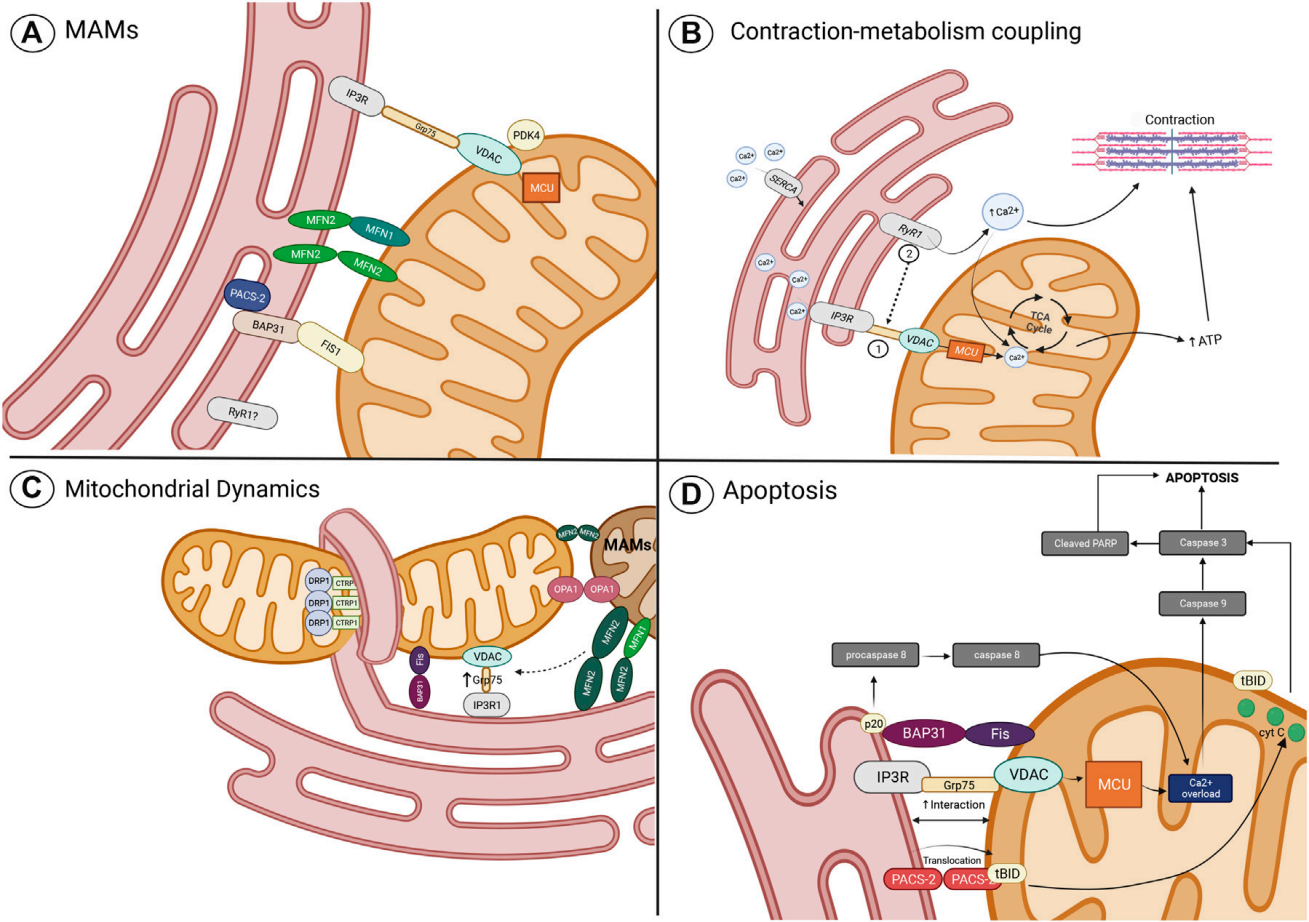
Role of MAMs in the regulation of cellular processes. **(A)** Main MAM proteins identified in skeletal muscle; **(B)** Contraction-metabolism coupling mediated by the transport of Ca^2+^ from SR/ER to mitochondria through the MAM complex, the IPR3-GRP75-VDAC-MCU, which function can be altered by the proposed mechanisms of ER stress and mitochondrial dysfunction; **(C)** role of proteins related to mitochondrial dynamics in the tethering of mitochondria and ER (or SR to skeletal muscle), calcium transfer, and mitochondrial fission and fusion regulation; and **(D)** MAMs proteins related to the apoptosis pathway. Imagen created with BioRender.com.

As mentioned before, the SM is recognized as high energy–demanding tissue, particularly due to the elevated ATP demand required during cell contraction and relaxation (Romanello and Sandri, 2022). Therefore, the mitochondrial sensing capacity of this energy demand is fundamental for an adequate SM function. The above is achieved by a well-established communication between the SR and mitochondria, the two main organelles that regulate contraction, relaxation, and energy production. According to numerous pieces of evidence, this communication is mediated by Ca^2+^, a key second messenger responsible for the regulation of ATP synthesis through the activity modulation of diverse TCA cycle enzymes, proteins, and transporters (Rossi et al., 2019). In this context, induced depolarization in SM has been related to mitochondrial metabolism stimulation given by Ca^2+^ transfer between the SR and mitochondria, an event described as contraction–metabolism coupling (Díaz-Vegas et al., 2018). The Ca^2+^ transfer from SR to mitochondria is one of the most relevant and well-studied mechanisms of MAMs. A decade ago, it was observed in SM that mitochondria are in proximity to the SR, particularly where the Ca^2+^ release units (CRUs) are located (Boncompagni et al., 2009). CRUs are interaction regions between the T tubules (TT), where L-type Ca^2+^ channels (LTCCs) are located in the sarcolemma and junctional domains of the SR which contain ryanodine receptors (RyRs), the main release Ca^2+^ channels of SR. LTCC of SM physically interacts with the RyRs; therefore, after membrane depolarization, the activated LTCC transmits the signal to RyRs, which in turn induces a massive Ca^2+^ release to the cytosol (Franzini-Armstrong et al., 1999). During postnatal maturation and the adult stage, a continuous increase in the number of CRUs, mitochondria, and CRU–mitochondria interactions was observed (Boncompagni et al., 2009). Therefore, it appears that the close distance between mitochondria and CRUs allows the generation of microdomains of high local Ca^2+^ concentration, which are required to activate the mitochondrial Ca^2+^ uptake (Boncompagni et al., 2009). Recently, in SM and other tissues, important participation of the protein complex IP3R-GRP75-VDAC was observed in the generation of the tethers and the mitochondrial Ca^2+^ uptake through the OMM (Tubbs et al., 2014, 2018; Thoudam et al., 2019). In addition, Ca^2+^ uptake through the IMM by the low-affinity MCU has also been suggested to be part of the IP3R-GRP75-VDAC1 complex (Patergnani et al., 2011; Arruda et al., 2014; Cheng et al., 2020; Carpio et al., 2021). In SM, it has been observed that the IP3R-GRP75-VDAC1 complex conformation is an important determinant for formation of MAMs and mt [Ca^2+^] signaling (Tubbs et al., 2018; Thoudam et al., 2019). An increase in IP3R-Grp75-VDAC1 complexes has been related to an increase in MAMs and mt [Ca^2+^] (Thoudam et al., 2019), while reduced expression of the IP3R-Grp75-VDAC1 by an induced stress condition or by a specific knockdown treatment has been related to a decrease in MAMs and mt [Ca^2+^] (Tubbs et al., 2018; Thoudam et al., 2019). Ca^2+^ transfer starts with the release of [Ca^2+^]_SR_ mediated by an SR Ca^2+^ channel. Therefore, it is to be expected that IP3R plays an important role in Ca^2+^ transfer. The IP3R is a Ca^2+^ channel mainly expressed in the SR, activated by IP3, and regulated by Ca^2+^ entry across the plasma membrane (Prole and Taylor, 2016). Besides the fast Ca^2+^ transients induced by RyR1 opening during ECC, an IP3R-induced [Ca^2+^]_SR_ release has been observed in SM, resulting in a long-lasting Ca^2+^ transient (Casas et al., 2010). This [Ca^2+^]_SR_ release has been related to cell contraction gene transcription regulation induced by cell depolarization (Casas et al., 2010), and Ca^2+^ transfer to mitochondria, and mitochondrial metabolism stimulation (Figure 2B) (Díaz-Vegas et al., 2018). Nevertheless, the role of IP3R in SM has been a topic of debate in the last two decades since other studies also showed a null effect of IP3R in the [Ca^2+^]_SR_ release (Blaauw et al., 2012). Therefore, it appears that the different roles shown by IP3R are related to their distinctive location and the diversity of IP3R isoforms expressed in SM (Casas et al., 2010). While IP3R2 is observed predominantly in clusters at the core of the fiber and in the perinuclear regions, IP3R3 is expressed in a striated pattern related to SR location, and IP3R1, the isoform observed in MAM fractions, is preferentially expressed in fast-type fibers with a characteristic mosaic pattern (Casas et al., 2010; Thoudam et al., 2019). In addition, it appears that IP3R1 is not the only Ca^2+^ channel that participates in the Ca^2+^ transfer from SR to the mitochondria. Recently, it has been described that RyR1 is also observed in the MAM fractions from SM (Thoudam et al., 2019), where immunoblot analysis, co-immunoprecipitation, and *in situ* proximity ligation assay also showed the presence of the IP3R1-GRP75-VDAC1 complex; nevertheless, physical interaction between RyR1 and the IP3R1-GRP75-VDAC1 complex not was found (Thoudam et al., 2019). RyR1 and IP3R have shown an important role in the Ca^2+^ transfer to mitochondria in SM since it has been observed that mt [Ca^2+^] increase after depolarization was completed and prevented only after the inhibition of the two SR Ca^2+^ channels (Díaz-Vegas et al., 2018). In this scenario, RyR1 and IP3R activation were related to basal oxygen consumption rate (OCR) and basal ATP-linked OCR. Nevertheless, only the RyR1 inactivation reduced the OCR and ATP-linked OCR after plasma membrane depolarization, suggesting that RyR1 has an important role in the Ca^2+^ transfer through MAMs during the contraction–metabolism coupling of SM (Figure 2B) (Díaz-Vegas et al., 2018). Nevertheless, most of the evidence of Ca^2+^ transfer through MAMs has been focused on the role of the IP3R-Grp75-VDAC complex; therefore, RyR1 functions as MAMs in SM need further exploration.

### Bidirectional regulation between mitochondrial dynamics and mitochondria-associated endoplasmic reticulum membranes and their role in skeletal muscle

A broader view of mitochondrial functions includes its ability to move and physically interact in dynamic reticular networks (Fealy et al., 2021). Mitochondria transition from punctuate structures to branched elongated tubules within the cell, preceding signaling events that influence various aspects of cellular functions (Romanello et al., 2010; Vincent et al., 2019). In response to changes in cellular metabolic demand, mitochondria adapt their morphology and content through a quality control system that ensures mitochondrial function (Sebastián et al., 2012). The preservation of the overall mitochondrial network morphology and integrity relies on the balance between mitochondrial fusion and fission processes known as mitochondrial dynamics; its coordination cycles with finely tuned mitochondrial turnover are regulated by biogenesis and mitophagy (Romanello and Sandri, 2022). Mitochondrial fusion is the process where mitochondria merge and aid in stress alleviation by diluting components of partially impaired mitochondria (Hemel et al., 2021; Wang et al., 2022). Fusion is mediated by the outer mitochondrial membrane-bound GTPases, Mitofusin 1 (Mfn1) and Mitofusin 2 (Mfn2), and the optic atrophy protein 1 (OPA1), a dynamin-like GTPase in charge of the inner membrane fusion (Romanello and Sandri, 2022). Fission is mediated by DRP1 and its adaptor proteins such as the mitochondrial fission factor (Mff), the mitochondrial fission one protein (Fis1), and the mitochondrial elongation factor 2/mitochondrial dynamics protein 49 (MIEF2/MiD49 and MIEF/1/MiD 51). Fission is a process that promotes the separation and segregation of dysfunctional mitochondria for selective elimination (Hemel et al., 2021; Romanello and Sandri, 2022; Wang et al., 2022). Mitochondrial dynamics is one of the fundamental ways in which cells achieve control over the population and function of their mitochondria under stress conditions, allowing mitochondria to respond and accommodate immediately to cellular stress (Wang et al., 2022). Current research suggests that the molecular machinery underlying the well-orchestrated processes of mitochondrial fusion and fission are coordinated at MAMs (Hemel et al., 2021; Wang et al., 2022). Mfn1 and Mfn2 can be localized in mitochondria, particularly in punctate structures where ER tubules cross the mitochondrial membrane–forming nodes. Mfn1 proteins that are localized to contact sites with the ER denote the location of mitochondrial fusion events (Abrisch et al., 2020). Similarly, the recruitment and assembly of DRP1 for activation of mitochondria fission requires the pre-constriction of MOM and MIM mediated by the interaction between mitochondria and ER, particularly, the envelopment of the mitochondrial membrane by ER at specific nodes (Friedman et al., 2011; Moore et al., 2016). Mfn2 has also been found in ER/SR membrane where forms homotypic interactions with Mfn2 located in MOM, or heterotypic interactions with Mfn1. Therefore, Mfn2 plays an important role as a tethering protein of MAMs (de Brito and Scorrano, 2008).

Recently, ER location of the cytosolic C1q/TNF-related protein 1 (CTRP1) has been associated with the recruitment of DRP1 at MAMs. CTRP1 is a cytosolic protein related to different roles in metabolic and cardiovascular functions through modulation of mitochondrial function (Sonn et al., 2021). A novel function of CTRP1 includes a role in the events required for mitochondrial fission to occur when this protein is located at the ER membrane. In this context, it has been found that CTRP1 ablation suppresses the recruitment of DRP1 to mitochondria and inactivates the fission event. Therefore, the localization of CTRP1 to the ER membrane and its recruitment and interaction with Drp1 to mitochondria has proven to be a critical event for fission (Sonn et al., 2021). Interestingly, Mfn1 and Drp1 colocalize and coordinate their functions (fusion and fission) at the same nodes of MAMs, observed in U-2 OS and HeLa cells, which suggests that mitochondrial morphology and quality control can be regulated at the same MAM positions, independently of the activated mechanism of mitochondrial dynamics (Figure 2C) (Abrisch et al., 2020). In this context, it has been observed that filamentous actin (F-actin) participates as an important regulator of fusion and fission events, by cycling from F-actin polymerization around mitochondria which induces fission and inhibits fusion, to F-actin depolymerization which allows the fusion of closer mitochondria. These F-actin polymerization–depolymerization cycles move from one mitochondria subpopulation to another, regulating the sites of mitochondria fission and fusion in this way (Moore et al., 2016).

The regulation of MAMs has also been observed to be influenced by the altered expression and function of the proteins that conform to the mitochondrial dynamics machinery. The expression or activity of OPA1 has been related to MAM regulation in fibroblasts and cells with lower expression of OPA1 due to mutation at the GTPase or GADE domain, which exhibited closer ER–mitochondria contacts accompanied by less Ca^2+^ transfer necessary to increase mt [Ca^2+^] (Cartes-Saavedra et al., 2022). Besides the fusion-related function of Mfn2, shown in Table 1, Mfn2 is a key regulator of the mitochondria–endoplasmic reticulum tethering observed *in vitro* (de Brito and Scorrano, 2008) and *in vivo* (Han et al., 2021). However, it appears that their function can be variable according to the cell type, the tissue, or the condition. In this context, in mouse embryonic fibroblasts, downregulation of Mfn2 promotes the increase in ER–mitochondrial contacts, leading to Ca^2+^ overload and cell death (Filadi et al., 2015). Meanwhile, in SM, in the *vastus lateralis* biopsies of healthy men, comparing interactions between subjects with a high abundance of Mfn2 versus those with a low abundance of it showed that the latter had a lower number of interactions (Castro-Sepulveda et al., 2021). In addition, in a model of amyotrophic lateral sclerosis, SM presented reduced content of Mfn2 and increased content of DRP1, compared with WT animals. These conditions were accompanied by reduced MAMs and an altered mitochondrial network, as observed in human biopsies with lower content of Mfn2 (Marini et al., 2020). This could be related to the fact that Mfn2 can cause diverse contact phenotypes in different paradigms, which may be determined by other tethering proteins (Eisner et al., 2018).

### Role of mitochondria-associated endoplasmic reticulum membranes on insulin signaling control and its relationship to insulin resistance development

As described above, mitochondrial dysfunction and ER stress are two key components of IR. In the past, they were studied independently; now, it is well recognized that both conditions are interrelated and accompany each other in different pathologies. In IR induced by HFD, mitochondrial dysfunction and ER stress were observed, accompanied by oxidative stress, JNK activation, and reduced expression of IRS1 and AKT (Yuzefovych et al., 2013). Despite the evidence, the causal mechanism of mitochondrial dysfunction and ER stress interaction and their role in IR is not well understood. ROS signal and Ca^2+^ have been identified as important routes of communication between ER and mitochondria. Therefore, the damage of one organelle can be transferred to the other through these mediators, initially promoting the activation of adaptive responses, but if the insult continues, it starts a maladaptive process that can lead to cell death (Csordás and Hajnóczky, 2009; Paillard et al., 2013; Honrath et al., 2017; Thoma et al., 2020). In this context, in SM mitochondrial dysfunction experimentally induced by altering the mitochondrial morphology (loss of OPA1) promotes an increase in ROS which in turn leads to activation of ER stress, leading to the induction of FGF21 expression, a hormone related to control of energy homeostasis, whose expression is related to IR (Pereira et al., 2017; Tezze et al., 2017). In ER, the pharmacologic induction of ER stress is also related to an increase in ROS that leads to altered mitochondrial function and morphology (Thoma et al., 2020). Altered expression of selenon, a selenoprotein N of the SR, whose loss of function leads to myopathy, is associated with IR development. Under lipid treatment, SM cells with loss of selenon function exhibit ER stress induced by lipotoxicity, induction of mitochondrial dysfunction, and altered MAMs (Varone et al., 2019). The physical and functional communication between ER and mitochondria is mediated by MAMs, which participate in the transfer of Ca^2+^ and ROS signals. Nevertheless, much remains to be explored in MAMs of SM, and it has not been ruled out that besides Ca^2+^ and ROS, other signal molecules may play important functions such as chaperons, cytokines, and nuclear and mitochondrial DNA, among others, affecting the development and course of diverse pathologies. Insulin susceptivity could be related to changes in MAMs due to alterations in the structure, signaling, and protein levels of MAMs that have been related to the development of IR in different tissues (Cheng et al., 2020), highlighting the relevance of these structures in the development of metabolic diseases. In this regard, it has been described that disruption of the ER–mitochondria interactions is an early event that precedes mitochondrial dysfunction and IR in SM. *In situ* gastrocnemius muscle evaluations of ob/ob and diet-induced IR in mice models showed a marked reduction in amount and interaction of MAMs (Tubbs et al., 2018). However, it is controversial due to the fact that it has also been evidenced that upregulation of MAM-related proteins leads to IR by increasing mitochondrial dysfunction induced by high Ca^2+^ transport to mitochondria mediated by MAMs (Arruda et al., 2014; Thoudam et al., 2019). On the other hand, increasing mitochondria–ER tethering by overexpression of Mfn2 seems to improve insulin sensitivity and GLUT4 expression and translocation to the plasma membrane (Kong et al., 2013) or by GRP75 by promoting VDAC–IP3R1 interactions in human myotubes (Tubbs et al., 2018). However, controversy has arisen from the fact that some studies suggest that increases in the interaction of mitochondria and the ER can inhibit insulin signaling. Pyruvate dehydrogenase kinases (PDKs) can regulate glucose utilization by being negative regulatory enzymes of the activity, *via* phosphorylation, of the pyruvate dehydrogenase (PDH) complex (Ma et al., 2020). PDKs are expressed in a tissue-specific manner; PDK4 is the main isoenzyme expressed in SM and the heart (Thoudam et al., 2019). In obesity-induced IR by an HFD and *ob/ob* mice, its expression is significantly higher. PDK4 interacts with the IP3R1-GRP75-VDAC1 complex and contributes to MAM formation in muscle; however, it also alters insulin signaling, while its suppression dampens MAM formation and downregulates JNK activation (Thoudam et al., 2019).

As mentioned before, ectopic lipid accumulation induced by increased FA uptake has been described as an important mechanism by which alterations in mitochondrial function might contribute to IR in SM (Jheng et al., 2012; Petersen and Shulman, 2018; Axelrod et al., 2021). Lipid overload can disrupt organelle coupling as was evidenced in palmitate-induced IR in human myotubes, where a significant reduction of VDAC1–IP3R1 interaction and a lower percentage of the mitochondrial membrane in contact with ER analyzed by TEM were notorious (Tubbs et al., 2018). In *vastus lateralis* biopsies of healthy subjects, respiratory quotient, a measure of relative whole-body lipid oxidation at rest and after exercise, was inversely related to IMF mitochondrial size, as well as to the percentage of mitochondria–sarcoplasmic reticulum associations, while mitochondrial size had an inverse association with lipid droplet density and a direct association with Mfn2/Fis1 ratio in this subpopulation (Castro-Sepulveda et al., 2020).

The study of MAMs and Ca^2+^ homeostasis associated with IR development has gained relevance in recent years. In this context, it is well established that alterations in the integrity of the MAMs due to changes in the expression of proteins that conform or stabilize the IP3R-Grp75-VDAC complex induced by conditions of obesity, T2D, HFD, high-sucrose diet, and genetic modification have been associated with IR development (Arruda et al., 2014; Tubbs et al., 2014, 2018; Thoudam et al., 2019). Nevertheless, controversial results have been observed, since some studies in SM and other tissues suggest that obesity is related to an increase in MAMs, which lead to a rise in mt [Ca^2+^] and mitochondrial dysfunction, which contributes to IR development (Arruda et al., 2014; Thoudam et al., 2019), while other studies found that obesity can also induce a reduction of MAMs and mt [Ca2+], conditions, which were also related to IR development (Tubbs et al., 2014; Tubbs et al., 2018; Zhang et al., 2020). It has been argued that the variability of the results obtained by different groups can be attributed to differences in the models used and the techniques developed for the analysis of the interaction between ER and mitochondria membranes (Thoudam et al., 2019). Nevertheless, it appears that regardless of the type of alteration observed in the MAMs (increase or disruption), the alteration of the Ca^2+^ signaling can lead to different consequences related to the IR in muscle.

### Transduction of apoptotic signals through mitochondria-associated endoplasmic reticulum membranes in skeletal muscle

The bidirectional transduction of cell stress signals between ER and mitochondria is a fundamental activity for cell homeostasis maintenance and death. ER is a key sensor of cellular stress signals, while mitochondria emit them to ER for signal amplification and respond to those sensed by ER, activating different mechanisms of cell death. Because the crosstalk of the stress signals between the two organelles is given by MAMs (Wang et al., 2021), it is to be expected that MAMs play an important role as a regulator of apoptosis. Different protein complexes related to MAM function have been explored in the apoptosis context. The IP3R-Grp75-VDAC protein complex has gained popularity due to its role in Ca^2+^ transfer between the ER and mitochondria and, therefore, due to their important contribution to apoptosis (Paillard et al., 2013; Honrath et al., 2017; Xu et al., 2018; Tiwary et al., 2021). On the one hand, it promotes metabolism as described in a previous section, and on the other hand, mt [Ca^2+^] overload can induce cell death. During diverse cellular stress conditions related to apoptosis, it has been observed that overexpression of the IP3R-Grp75-VDAC complex proteins and the MCU increases their interaction and, therefore, augments tethering between the ER and mitochondria with the Ca^2+^ overload as a common consequence (Paillard et al., 2013; Honrath et al., 2017; Xu et al., 2018; Tiwary et al., 2021). In this scenario, Ca^2+^ overload has been related to elevated ROS production, mitochondrial dysfunction, elevated apoptosis rate, caspase-3 activation, and enhanced cleaved PARP levels (Paillard et al., 2013; Xu et al., 2018; Tiwary et al., 2021). Conditions prevented by the action of antagonists or downregulation of IP3R, Grp75, VDAC, or MCU (Paillard et al., 2013; Honrath et al., 2017; Xu et al., 2018; Tiwary et al., 2021). In addition, different molecules participate in the regulation of apoptosis through interaction with MAM proteins, particularly, regulating the function of the IP3R and Ca^2+^ transfer (Wang et al., 2021). Inhibitory phosphorylation of IP3R by AKT reduces Ca^2+^ transfer to mitochondria and cell apoptosis, and IP3R activity can also be indirectly modulated by the PLM and PTEN proteins, which regulate the AKT activity (Wang et al., 2021). The impact of the IP3R-Grp75-VDAC complex in apoptosis has been explored in podocytes, pancreatic insulinoma cells, primary islet cells, hippocampal neuronal cells, cardiomyoblasts, and adult cardiomyocytes (Paillard et al., 2013; Honrath et al., 2017; Xu et al., 2018; Tiwary et al., 2021). In SM, similar enhanced MAMs due to the IP3R1-Grp75-VDAC protein’s overexpression and augmented interaction have been found in conditions of IR and obesity, where mt [Ca^2+^] overload has also been found (Thoudam et al., 2019). The relation between IP3R-Grp75-VDAC and apoptosis in SM has not been explored yet. Nevertheless, apoptosis of SM with enhanced caspase-3 activation and cleaved PARP have been found to be induced by an HFD in a model of obesity (Sishi et al., 2011). In addition, blocking or knockdown of IP3R1 in a neuromuscular junction Ca^2+^ overload model prevented the activation of caspase-3 and caspase-9 (Zhu et al., 2011). Therefore, it is expected that the IP3R1-Grp75-VDAC complex proteins and regulator molecules seen in other tissues play an important role in the regulation of SM apoptosis under physiological and pathological conditions, which requires a more comprehensive evaluation.

Besides the implication of IP3R1-Grp75-VDAC complex in MAM transduction of stress signals and apoptosis activation, other proteins have gained relevance in different tissues. Nevertheless, very little is known about SM and IR. Phosphofurin acidic cluster sorting protein 2 (PACS-2) is a sorting protein that participates in multiple cell functions (Li et al., 2020). PACS-2 was one of the first proteins identified as a MAM formation regulator (Simmen et al., 2005) and has been found to be overexpressed in obesity, associated with an increase in MAM formation and Ca^2+^ overload in the liver (Arruda et al., 2014). PACS-2 is an important apoptosis regulator, where their pro-apoptotic or anti-apoptotic functions are related to the cell type and the stimulus (Li et al., 2020). A diversity of apoptotic inducers stimulates the translocation of PACS-2 from ER to mitochondria, where PACS-2 recruits the proapoptotic factor BH3-only Bcl-2 family member, Bid. In mitochondria, Bid is cleaved to tBid which stimulates the cyt-c release, and the subsequent activation of caspase-3 leads to cell death (Simmen et al., 2005). Similarly, the TRAIL-induced apoptosis pathway which kills diseased cells such as tumor cells or virus-infected cells can be mediated by PACS-2, where dephosphorylation of PACS-2 triggered by TRAIL promotes their recruitment and translocation of Bid to mitochondria with the subsequent cyt-c release (Aslan et al., 2009). In SM, it has been observed that PACS-2 is highly expressed (Simmen et al., 2005); therefore, it is expected that PACS-2 plays an important role in the regulation of MAMs and apoptosis in SM, which requires further exploration. The Fis1-BAP31 complex is another key component of MAMs with important participation in the transduction of apoptotic signals. Fis1 and BAP31 form a bridge that allows the transfer of apoptotic signals from mitochondria to ER. BAP31 is a protein chaperone located in the ER membrane; it physically interacts with FIS1, which induces the cleavage of BAP31 to the pro-apoptotic p20 BAP31. p20 BAP31 recruits and activates pro-caspase-8, promoting ER release of Ca^2+^ and Ca^2+^ transfer to mitochondria for signal amplification, with the subsequent activation of caspase-3 and cell death (Iwasawa et al., 2011; Wang et al., 2021). The BAP31 expression can vary according to the type of tissue; it is mainly expressed in brain and immune cells, while low expression of BAP31 is observed in muscle (Quistgaard, 2021). Therefore, the role of the FIS1-BAP31 complex in the transduction of apoptotic signals may have a minor implication on the function of MAMS in muscle (Figure 2D).

### Modulation of mitochondria-associated endoplasmic reticulum membranes as treatment targets

It is evident that MAMs have a role in a great number of functions and that miscommunication between mitochondria and the ER leads to important alterations; therefore, they represent a platform where strategies that can improve glucose homeostasis could be of benefit (Arruda et al., 2014). As mentioned before, it is still not clear whether increasing contact by overexpression of specific MAM proteins (Kong et al., 2013; Tubbs et al., 2018) or downregulation is the best strategy to restore the alterations in insulin signaling (Arruda et al., 2014; Cheng et al., 2020). The evidence suggests that the best strategy is to maintain balance in the MAMs. In this sense, there are numerous studies where interventions such as exercise or diet changes have been proposed to restore the energy imbalance caused by obesity and, in many cases, reverted IR (Merle et al., 2019; Miller et al., 2020; Ryan et al., 2020; Grevendonk et al., 2021; Mizunoe et al., 2021). Therefore, exercise could be a good strategy to maintain or restore balance at the MAM level. Additionally, exercise also changes the proportion of fibers in SM; under obesity or IR conditions, type II fibers are the most abundant but could be reverted by exercise and, consequently, MAMs and insulin sensitivity (Fry, 2004; Qaisar et al., 2016; Blocquiaux et al., 2020). Therefore, with what is described here, those interventions could be a good strategy to restore the MAM alterations induced by lipotoxicity and thus reestablish contractility alterations, mitochondrial dysfunction, and insulin sensitivity in the SM.

## Conclusion and future perspectives

In mammalian cells, oxidative insults activate stress response pathways including inflammation, cytokine secretion, and apoptosis. Intriguingly, mitochondria are emerging as a sensor network that may function as an early indicator of subsequent cellular stress responses. A new perspective of mitochondrial dysfunction is defined as the altered capacity of mitochondria to communicate with other organelles within IR tissues such as lipid droplets, Golgi apparatus, lysosomes, and peroxisomes (Keenan et al., 2020). Although the characterization of different organelles has long been the subject of many studies, research on how these distinct compartments communicate is a more recent phenomenon (Pichla et al., 2021). Inter-organelle communication in the pathogenesis of mitochondrial dysfunction and IR is a mechanism that requires more study, due to organelle interactions being temporally and spatially formed connections essential for normal cell function. Despite a growing body of work related to understanding these organelle contacts, the changes in mitochondria–ER communication in obesity and IR require further investigation. Conflicting evidence warrants further research in understanding ER–mitochondria contact sites and particularly addresses whether disruption of this process causes IR or is a consequence of pre-existing IR perturbations in mitochondrial contacts that may lead to metabolic dysfunction and support the notion that modulating organelle interactions could be a promising strategy for the treatment of metabolic disease. Future studies will need to explore this possibility. We propose that there could be a differential interaction between the main mitochondrial subpopulations and the ER depending on the type of fibers. Besides proposing MAMs as therapeutic targets for interventions that affect excitation–metabolism coupling such as dietary and physical activity intervention, understanding communication between mitochondria and the ER in SM would allow us to understand the beneficial role of MAM control, which is still controversial.
